# Proton Magnetic Resonance Spectroscopy Biomarkers in Neonates With Hypoxic-Ischemic Encephalopathy: A Systematic Review and Meta-Analysis

**DOI:** 10.3389/fneur.2018.00732

**Published:** 2018-08-31

**Authors:** Rong Zou, Tao Xiong, Li Zhang, Shiping Li, Fengyan Zhao, Yu Tong, Yi Qu, Dezhi Mu

**Affiliations:** ^1^Department of Pediatrics, West China Second University Hospital, Sichuan University, Chengdu, China; ^2^Key Laboratory of Birth Defects and Related Diseases of Women and Children, Ministry of Education, Sichuan University, Chengdu, China

**Keywords:** proton magnetic resonance spectroscopy, hypoxic-ischemic encephalopathy, therapeutic hypothermia, neurodevelopmental outcome, prognostication

## Abstract

**Background:** Hypoxic-ischemic encephalopathy (HIE) is a major contributor to child mortality and morbidity. Reliable prognostication for HIE is of key importance. Proton magnetic resonance spectroscopy (^1^H-MRS) is a quantitative, non-invasive method that has been demonstrated to be a suitable complementary tool for prediction. The aim of this study was to investigate the prognostic capability of ^1^H-MRS in the era of therapeutic hypothermia (TH).

**Methods:** Databases, namely MEDLINE, Embase, Web of Science, and the Cochrane library (Cochrane Center Register of Controlled Trials), were searched for studies published before July 17, 2017. Study selection and data extraction were performed by two independent reviewers. The mean difference (MD) or standardized MD (SMD) and 95% confidence interval (CI) were calculated using random-effects models. Subgroup analyses were conducted based on the use of TH.

**Results:** Among the 1,150 relevant studies, seven were included for meta-analysis, but only two small studies were conducted under TH. For ^1^H-MRS measurement, three peak area ratios revealed predictive values for adverse outcomes in TH subgroup and the combined results (with and without TH): N-acetylaspartate (NAA)/creatine in basal ganglia/thalamus (BG/T) in TH (MD −0.31, 95%CI −0.55 to −0.07) and combined results (MD −0.37, 95% CI −0.49 to −0.25); NAA/choline in BG/T in TH (MD −0.89, 95%CI −1.43 to −0.35) and combined results (MD −0.25, 95%CI −0.42 to −0.07); and myo-inositol/choline in cerebral cortex in TH (MD −1.94, 95%CI −3.69 to −0.19) and combined results (MD −1.64, 95%CI −2.64 to −0.64). Moreover, NAA relative concentration is associated with adverse outcomes: in TH (MD −0.04, 95%CI −0.06 to −0.02) and combined results (MD −0.06, 95%CI −0.11 to −0.01) in white matter; in TH (MD −0.04, 95%CI −0.07 to −0.01) and combined results (MD −0.05, 95%CI −0.07 to −0.02) in gray matter.

**Conclusions:** NAA may be a potential marker in outcome prediction for all HIE subjects. It seems that MDs for the ratios including NAA are larger than for its relative concentration, and therefore are more likely to be measurable in a clinical context. Larger prospective multicenter studies with a standardized protocol for both measurement protocols and analysis methods are required in future studies.

## Introduction

Hypoxic-ischemic encephalopathy (HIE) is a leading worldwide contributor to child mortality and life-long morbidities and occurs in ~1.5 per 1,000 live births (1). Despite the recent widespread use of therapeutic hypothermia (TH), 45–50% of these infants still experience adverse outcomes (2). In these critically ill neonates, reliable neurological prognostication is important. It can be referred to not only for appropriate clinical management decisions, but also rational parental counseling regarding the need for developmental interventions after discharge. Furthermore, a dependable evidence-based prognosis can serve as a useful surrogate endpoint as well as a bridging biomarker, which enables more effective and rapid evaluation and translation of novel synergistic therapies from laboratory to bedside (3–5).

In the past decades, studies investigating predictors of long-term neurodevelopmental outcomes, such as clinical neurological examination, serum biomarkers, neurophysiology, and advanced neuro-imaging modalities, have attained a significant role (6, 7). Among these tests, proton magnetic resonance spectroscopy (^1^H-MRS) is a quantitative, non-invasive method of detecting energy metabolism disturbances in the brain. Studies have demonstrated that deep gray matter (GM) lactate/N-acetylaspartate (Lac/NAA) may serve as a suitable complementary tool for prognostication (5, 8–10). It has already been used as a surrogate endpoint in a recent randomized controlled trial investigating xenon as a combined therapy (11).

However, in the wake of the era of TH, the capability of ^1^H-MRS to discriminate neonates with adverse outcomes from those with good outcomes may be altered. Recent studies have reported that TH exerts a significant influence on cerebral energy metabolism (12, 13). Therefore, the accuracy and reliability of ^1^H-MRS to predict the prognosis of HIE after TH needs to be reassessed. This systematic review of the published literature aimed to determine the extent to which TH impacts brain metabolism and the prognostic capability of ^1^H-MRS.

## Methods

This systematic review was performed according to the Preferred Reporting Items for Systematic Review and Meta-Analysis: the PRISMA statement (14).

### Search strategy

A literature search of databases, including MEDLINE, Embase, Web of Science, as well as the Cochrane library (Cochrane Center Register of Controlled Trials), for studies published in English before July 17, 2017, without a limit on the start date, was performed using Medical Subject Heading terms together with the following keywords: [Magnetic resonance spectroscopy OR MRS OR MR spectroscopy] AND [Hypoxic Ischemic Encephalopathy OR Asphyxia Neonatorum OR asphyx^*^ OR sarnat OR HIE]. A manual review of bibliographies and references from the retrieved articles was performed to further complement the search.

### Study selection

Relevant articles were screened by two authors independently and in duplicate to assess study eligibility. Studies were included if they met the following criteria: (1) ^1^H-MRS was performed during the neonatal period in infants with perinatal asphyxia and HIE; (2) at least a single metabolite ratio or concentration was presented for each group; (3) encephalopathic infants with normal long-term outcomes as control subjects were used; (4) neurodevelopmental outcomes were provided at 12 months of age or later, and defined clearly as adverse or good; and (5) inclusion and exclusion criteria for the infants enrolled in the study were identified.

Studies in which the subjects were animals, or the literature type was not “article,” and those that enrolled infants with congenital malformations, infections, or comorbid diseases were excluded. The study was also excluded if there was no sufficient data to obtain significant effect sizes. Discussion with a third reviewer was used to resolve disagreements.

### Data extraction

Data were extracted from the selected studies by two independent reviewers. Neurodevelopmental outcomes were recorded as either “adverse” (i.e., moderate/severe disability or death) or “good” (i.e., normal or mild disability) as defined in each study. When neurodevelopmental outcomes were assessed more than once, the most recent data from the follow-up were used for analysis. Values of important parameters of MRS detection were extracted, including those of metabolite peak area ratios, such as lactate/N-acetylaspartate (Lac/NAA), N-acetylaspartate/creatine (NAA/Cr), and N-acetylaspartate/choline (NAA/Cho), myo-Inositol/Cr (mI/Cr), and myo-Inositol/choline (mI/Cho), in each region of interest (ROI) respectively, which have been proposed to be potential predictive biomarkers in previous studies. The values of absolute or relative concentration of each metabolite included were also extracted, if possible. To ensure sufficient power in the meta-analysis, brain region measures were included if there were at least two eligible studies reporting ≥2 ROIs in total. For studies that indicated the ROIs precisely, the subregion was sorted into basal ganglia/thalamus (BG/T) and cerebral cortex, consistent with the common patterns of HIE injury under different pathological circumstances (15). Otherwise, subgroups were classified according to the tissue type contained in the ROIs, such as GM and white matter (WM) (16, 17). For studies in which data were separated into severe disability and death, pooled data were used in the analysis.

### Quality assessment

The quality of each study was assessed using the Quality Assessment of Diagnostic Accuracy Studies-2 (QUADAS-2), adapted for this particular review according to a previous study (18). The QUADAS-2 form comprises four domains, namely patient selection, index test, reference standard together with flow, and timing. For each domain, the risk of bias and concerns regarding applicability were analyzed, and rated as low risk, high risk, and unclear risk (19). Two authors assessed quality independently, using a predefined form containing the quality assessment criteria (Table S1), and disagreements were resolved through discussion.

### Statistical methods

All meta-analyses were performed using Review Manager 5.3 (Cochrane Collaboration) in a random-effects model. Continuous data, such as peak area ratios and relative concentration, were compared using mean difference (MD) and 95% confidence interval (CI). For comparison of absolute concentration, the standardized MD (SMD) and 95% CI were used to account for the use of different metrics. Heterogeneity was assessed using the I^2^ statistic, in which a heterogeneity of 0% was considered negligible, 0–20% minimal, 20–50% moderate, and >50% was deemed substantial. Subgroup analyses based on the use of TH were also conducted. The reliability of results was further tested using sensitivity analysis in a cyclic manner. More specifically, single studies were eliminated one at a time and the analysis was performed again to determine whether the results were stable.

## Results

### Study selection

**Figure 1:**
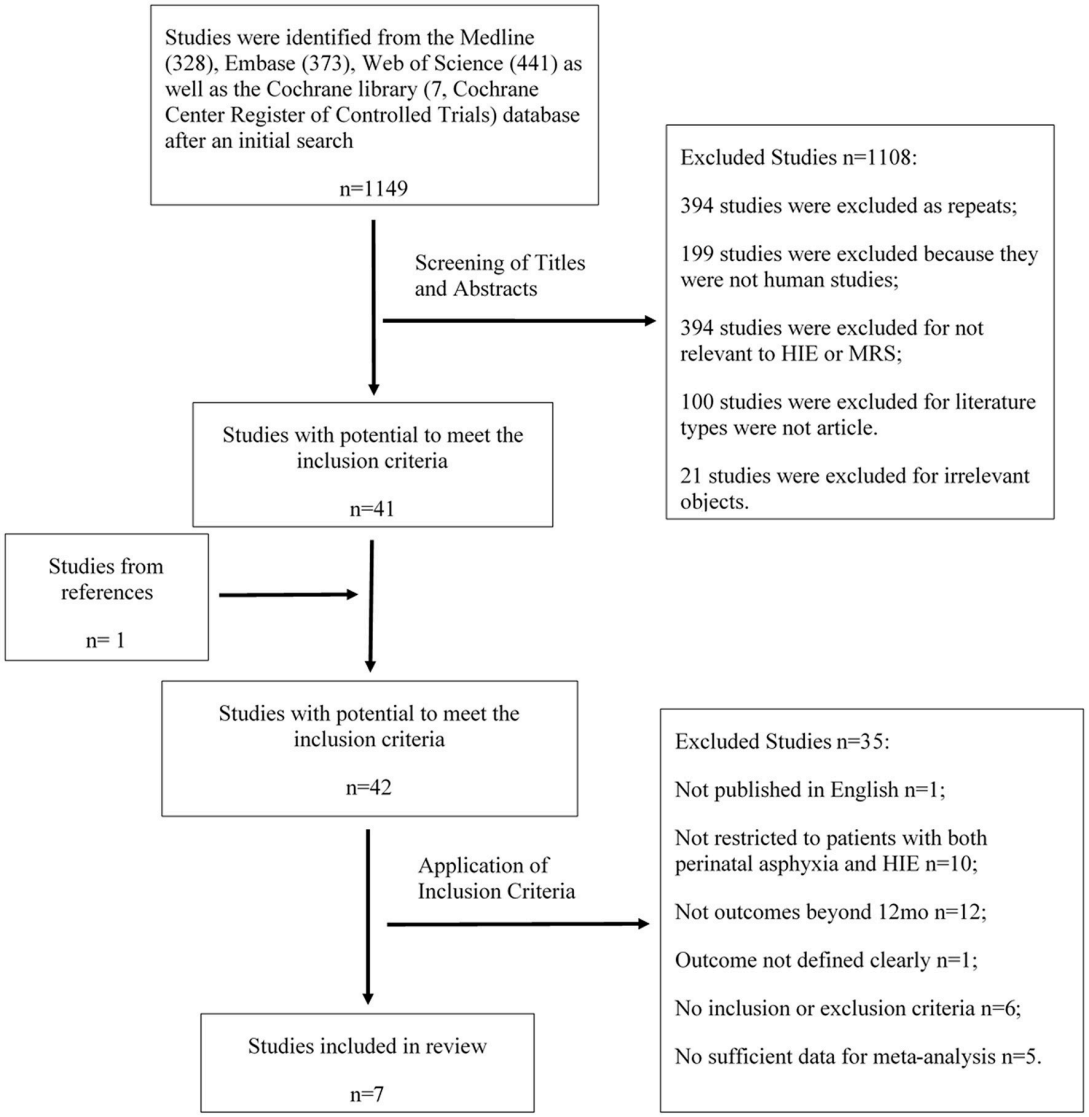
Study selection process. The flow chart shows the procedure of literature search and study inclusion and reasons for exclusion.

A total of 1,149 articles retrieved in the literature search were screened by two independent reviewers. More than 50% of studies were excluded, either because they were not human studies or were duplicates. Others were excluded because of irrelevant objects or non-eligible article types. Forty-one articles, in addition to 1 additional study selected by verifying citations, appeared to have the potential to meet the inclusion criteria. The full texts of these 42 studies were subsequently reviewed by two independent reviewers for final determination. Among these, 1 study was excluded because it was not published in English, 10 were rejected because they were not restricted to patients with both perinatal asphyxia and HIE, 12 provided neurodevelopmental outcomes within 12 months, 1 did not define the outcomes clearly, 6 lacked inclusion and/or exclusion criteria for the enrolled neonates, and 5 reported insufficient data for meta-analysis. Seven studies were finally included in the present review (Figure 1).

### Characteristics of included studies

The included studies reported ^1^H-MRS biomarkers of three different forms in several ROIs. Two studies reported relative concentration of metabolites instead of absolute quantification, which means that metabolites were expressed in percent (%) of summed peak areas of Cho, Cr, and NAA in all the analyzed voxels (16, 17). Two studies were restricted to neonates who underwent TH (16, 20). The characteristics of all 7 included studies are summarized in Table 1.

**Table 1:** Characteristics of included studies.

**Study**	**References**	**Country**	**Study design**	**N1/N2**	**Blinded (Y/N)^a^**	**TH (Y/N)^b^**	**Test**	**Sarnat stage (1/2/3)^c^**	**Male/Female**	**GA, wk**
1	Sijens et al. (16)	Netherlands	Retrospective	35/35	NA	Y	Relative concentration	NA	NA	≥36
2	Ancora et al. (20)	Italy	Prospective	20/20	NA	Y	Peak-area ratio	0/16/4	12/8	Term: 39.3 ± 1.3
3	van Doormaal et al. (17)	Netherlands	Retrospective	24/24	Y	N	Relative concentration	1/16/7	14/10	36–42
4	Ancora et al. (21)	Italy	Prospective	31/32	Y	N	Peak-area ratio.	17/13/2	24/8	≥37: 39.5 ± 1.3
5	Boichot et al. (22)	France	Retrospective	30/30	Y	N	Peak-area ratio; Absolute concentration	NA	12/18	>37
6	Cheong et al. (23)	UK	Prospective	17/17	NA	N	Peak-area ratio; Absolute concentration	5/5/7	NA	Term: 39.0 ± 1.6
7	Kadri et al. (24)	USA	Prospective	33/33	Y	N	Peak-area ratio	NA	15/18	38–42

*N1 indicates the number of cases included in review; N2 represents the total number of cases studied; NA, not available; GA, gestational age*.

a *Blinded (Y/N) shows if neurodevelopmental outcomes were assessed blinded to the proton magnetic resonance spectroscopy results*.

b *TH (Y/N) shows whether the enrolled infants were receiving therapeutic hypothermia treatment*.

c *Sarnat Stage (1/2/3) indicates the numbers of neonates in stage 1, 2, and 3 according to Sarnat Scores, respectively*.

Information regarding age at follow-up and tools used to evaluate outcomes is listed in Table 2, as well as the inclusion criteria and the definition of adverse outcomes. Five (71%) studies used the Bayley Scales of Infant Development and/or the Griffiths Mental Developmental Scale for outcome assessment, while 4 (57%) performed follow-up for at least 18 months' postnatal age. In 4 of the 7 studies (57%), the outcome assessors were blinded to the ^1^H-MRS results. Detailed information regarding the MR scanners and sequences used for ^1^H-MRS biomarkers is listed in Table 3.

**Table 2:** Study inclusion criteria and outcome measures.

**Study**	**References**	**Inclusion criteria^a^**	**Age at follow-up, mo**	**Outcome studied**	**Adverse outcomes**
1	Sijens et al. (16)	(1) Perinatal asphyxia (≥1): 5-min Apgar ≤ 5, or need for resuscitation/ventilation, or pH < 7, or BD>16 mmol/L, or lactate>10 mmol/L; (2) Clinical encephalopathy: Thompson>7 (1–3 h postpartum)	12–30	BSID-III; GMFCS	(1) Death; (2) BSID-III < 70; (3) CP with a GMFCS>3
2	Ancora et al. (20)	(1) Perinatal hypoxia-ischaemia: 10 min-Apgar ≤ 5, need for resuscitation, cord or early arterial/venous pH < 7.00 or BD≥16 mmol/L; (2) Clinical encephalopathy: Sarnat 2 and 3	24	GMDS; Sensorineural examinations	(1) Death; (2) CP; (3) DQ < 88.7; (4) Sensorineural deficits: like hearing or visual deficits
3	van Doormaal et al. (17)	(1) Perinatal asphyxia (≥2): decelerations or meconium-stained liquor, UA pH < 7.1; 5-min Apgar < 5; or multiorgan failure; (2) Clinical encephalopathy: Sarnat	≥18	BSID-II; GMFCS; Neurological examination	(1) Death; (2) GMFCS≥3
4	Ancora et al. (21)	(1) Perinatal asphyxia (all): fetal heart rate abnormalities and/or meconium-stained liquor and/or need for birth resuscitation, 5-min Apgar ≤ 5 and/or UA pH < 7.1 with BE≥12 mmol/L; (2) Clinical encephalopathy: Sarnat	24	GMDS; Sensorineural examinations	(1) Death; (2) CP; (3) DQ < 85; (4) Sensorineural deficits: hearing loss or cortical visual impairment
5	Boichot et al. (22)	(1) Perinatal asphyxia (≥2): (a) intrapartum fetal distress: fetal heart rate abnormalities or meconium-stained liquor, (b) neonatal distress: 5-min Apgar < 5, UA pH < 7.1, or need for immediate resuscitation; (c) organ dysfunction; (2) Clinical encephalopathy: Sarnat 2 and 3	12–82	Neurologic examination	(1) Death; (2) Severe disability as defined by World Health Organization criteria
6	Cheong et al. (23)	(1) Perinatal asphyxia (≥1): 1 min-Apgar < 5, cord or UA pH < 7.1, and/or BD>12 mmol/L, late decelerations, need for resuscitation at birth; (2) Clinical encephalopathy: altered conscious state, abnormal tone, and reflexes, feeding difficulties, and seizures (graded by Sarnat)	12	Modified Amiel-Tison assessment; GDS	(1) Death; (2) Major impairment with disability on neurologic assessment; (3) DQ < 75
7	Kadri et al. (24)	(1) Perinatal asphyxia (all): initial arterial pH < 7.15, 5 min-Apgar < 5; (2) Clinical encephalopathy: generalized hypotonia, lethargy, poor sucking and feeding, and respiratory failure	24	PCPCS	(1) Death; (2) Persistent vegetative state; (3) Severe disability as defined by PCPCS (conscious, dependent on others for daily support)

a *Inclusion Criteria, (≥n) demonstrates that the diagnosis was defined by at least n of the following criteria*.

*BD, base excess; BE, base deficit; BSID, Bayley scales of infant development; CP, cerebral palsy; DQ, development quotient; GDS, the Griffiths' Developmental Scale; GMDS, the Griffiths' Mental Developmental Scale; GMFCS, the Gross Motor Function Classification System; PCPCS, the Pediatric Cerebral Performance Category Scale; UA, umbilical artery*.

**Table 3:** Details of proton magnetic resonance spectroscopy biomarkers.

**Study**	**References**	**Age at Scan, d**	**Sedation (Y/N)^a^**	**ROI**	**MR specifications**	**Biomarker details**	**Abnormal findings**
1	Sijens et al. (16)	3–13	NA	Cranial to the CC (WM/GM)	1.5 Tesla Siemens AG with an 8-channel transmit/receive head coil	CSI (TR/TE 1,500/135 ms), MVS, 2 cm3 voxel	WM: Cr↑: gross motor scores↑ (*r* = 0.58, *P* = 0.02); Cho↑: fine motor scores↓ (*r* = −0.40, NS); GM: NAA↓; Cho↑: gross score↓ (*r* = −0.45, NS); NAA↑: cognitive scores↑ (*r* = 0.37, NS)
2	Ancora et al. (20)	4–16	Y	BG; P-O cortex; F-P WM	1.5 Tesla GE whole-body scanner, using a 25-cm diameter quadrature birdcage head coil	PRESS (TR/TE 1,500/40 ms), SVS, 5.1/4.6/3.9 cm3 voxel	BG: NAA/Cr ≤ 0.67, NAA/Cho ≤ 1.82; mI/Cr ≤ 0.76, mI/Cho ≤ 2.04; LL/NAA≥3.33 P-O cortex: NS F-P WM: NS
3	van Doormaal et al. (17)	3–16	NA	Cranial to the lateral ventricles (WM/GM)	1.5 Tesla Siemens AG with an 8-channel transmit/receive head coil	CSI (TR/TE 1,500/135 ms), MVS, 2 cm3 voxel	WM: NAA↓, Lac↑, Cho (NS), Cr (NS); GM: NAA↓, Lac↑, Cho↑, Cr (NS)
4	Ancora et al. (21)	7–10	Y	BGT; P-O cortex	1.5 Tesla GE whole-body magnet	PRESS (TR/TE 1,500/40 ms), SVS, (3.1–6.3)/4.5 cm3 voxel	BG: NAA↓, NAA/Cr↓, NAA/Cho↓ Lac(NS), Lac/Cr (NS), Lac/Cho (NS); mI↓, mI/Cr (NS), mI/Cho (NS) PO cortex: NAA↓, NAA/Cr < 0.5, NAA/Cho↓; Lac↑, Lac/Cr>0.3, Lac/Cho↑; mI>3.0 mM, mI/Cr (NS), mI/Cho↓
5	Boichot et al. (22)	2–12	N	BG; Frontal cortex; Parietal cortex; Occipital cortex	1.5 Tesla Siemens scanner	CSI (TR/TE 1,500/270 and 80 ms), MVS, 1 mL voxel	BG: NAA < 4 mmol/L, Cho↓, Cr (NS), NAA/Cho↓, NAA/Cr↓, Lac/Cho↑; Frontal Cortex: NAA↓, Cho↓, Cr↓, NAA/Cho (NS), NAA/Cr (NS), Lac/Cho (NS); Parietal Cortex: NAA↓, Cho↓, Cr↓, NAA/Cho↓, NAA/Cr↓, Lac/Cho↑; Occipital Cortex: NAA↓, Cho↓, Cr (NS), NAA/Cho↓, NAA/Cr↓, Lac/Cho (NS); Frontal WM: NAA↓, Cho↓, Cr (NS), NAA/Cho (NS), NAA/Cr ↓, Lac/Cho (NS); Occipital WM: NAA↓, Cho↓, Cr↓, NAA/Cho↓, NAA/Cr↓, Lac/Cho↑
6	Cheong et al. (23)	1–3	Part Y.	Thalami	2.4 Tesla Bruker Advance system with a head coil of 15-cm diameter and length	PRESS (TR/TE 2,000/270 ms), SVS, 8 mL voxel	Peak-area ratios: Lac/Cr (NS), Lac/NAA ↑ , NAA/Cr↓, NAA/Cho↓, Cho/Cr (NS); Absolute concentration: Lac (NS), NAA↓, Cho (NS), Cr (NS)
7	Kadri et al. (24)	2–12	NA	Occipital GM	1.5 Tesla Siemens whole-body imaging system	STEAM (TR/TE 3,000/20 ms), SVS, 8 cm3 voxel	Lac (presence)↑; NAA/Cr (NS), NAA/Cho↓; Cho/Cr (NS)

a *Sedation (Y/N) indicates whether the neonates were sedated or not before proton magnetic resonance spectroscopy examinations*.

*BG/T, basal ganglia or thalamus; CC, corpus callosum; Cho, choline;Cr, creatine; CSI, chemical-shift imaging; F-P, frontal-parietal; GM, gray matter; LL, lactate and lipids; LN, lentiform nucleus; mI, myo-inositol; MVS, multi-voxel spectroscopy; NA, not available; NAA, N-acetylaspartate; NS, not significant; P-O, parietal-occipital; PRESS, point-resolved spectroscopic sequence; ROI, region of interest; STEAM, stimulated-echo amplitude mode spectroscopy; SVS, single voxel spectroscopy; TE, echo time; Lac, lactate; TR, repetition time; WM, white matter*.

### Quality assessment

**Figure 2:**
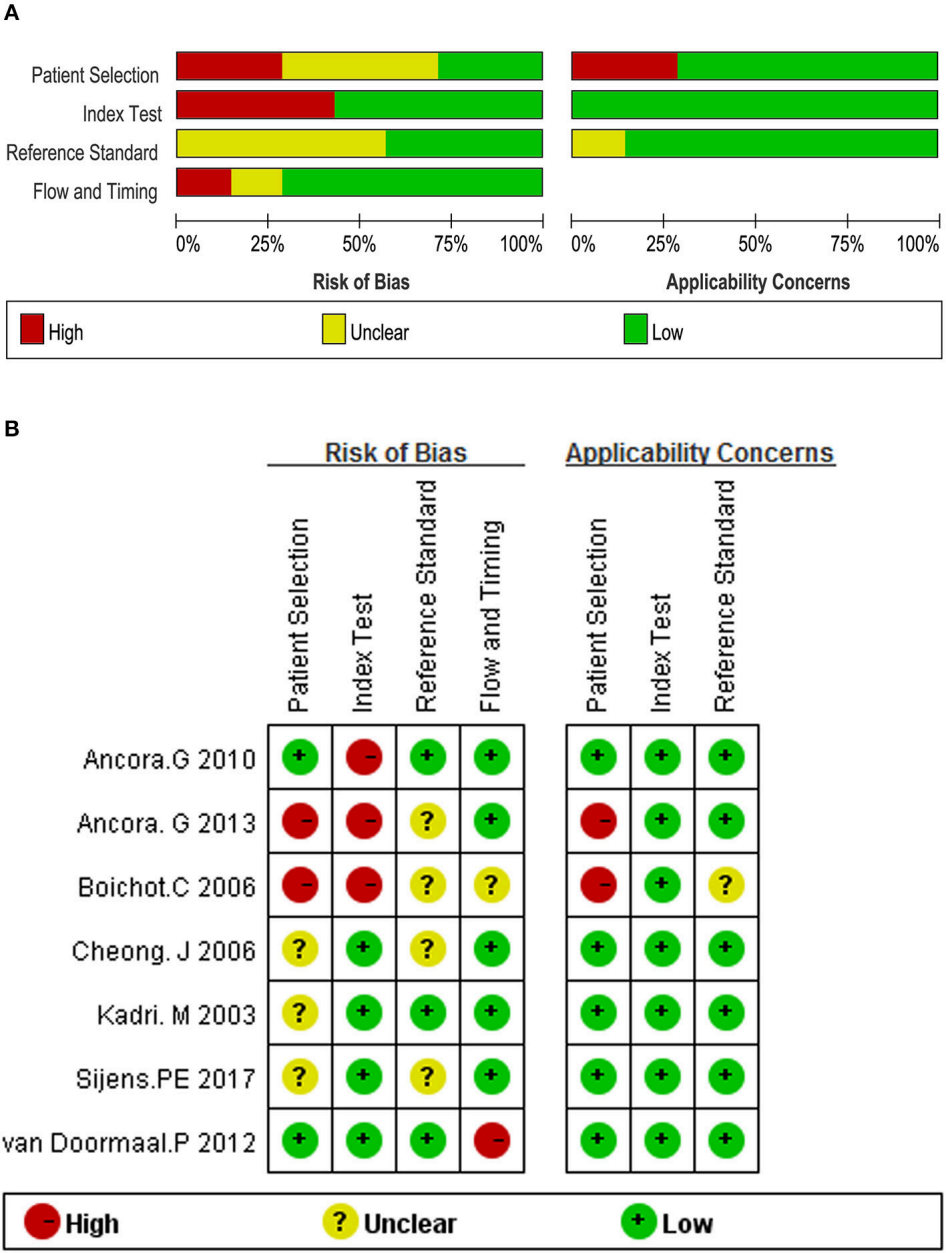
Quality assessment according to QUADAS-2 of the included studies **(A)** overall and **(B)** by study.

The overall evaluation of the risk of bias and applicability concerns is shown in Figure 2A. One of the major problems was the poor reporting of patient selection, as 4 (57%) studies did not report precisely whether the patients were selected consecutively or not. And 2 (29%) studies were ranked as high risk because they only included neonates with moderate to severe HIE. With respect to the domain of index test bias, 3 (43%) studies were rated as high risk because of the use of threshold deriving from the results of the study. The problem of poor reporting also existed in the domain of reference standard bias, in which 4 (57%) of the 7 studies did not explicitly report whether the outcome assessments were performed and interpreted without the knowledge of the ^1^H-MRS results. The details of each individual study are listed in Figure 2B.

### Meta-analysis

**Figure 3:**
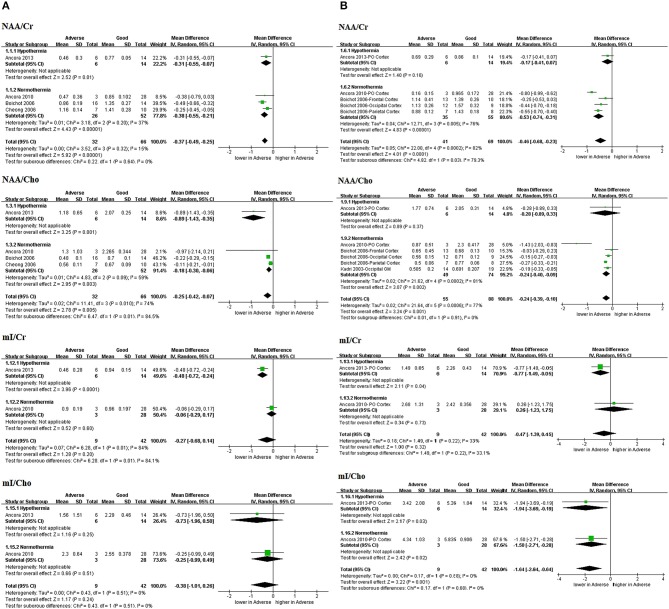
Graphic depiction of the results of meta-analyses: peak area ratios in the **(A)** BG/T and **(B)** cerebral cortex (NAA/Cr, NAA/Cho, mI/Cr, and mI/Cho). BG/T, basal ganglia or thalamus; Cho, choline; Cr, creatine; mI, myo-Inositol; NAA, N-acetylaspartate.

**Figure 4:**
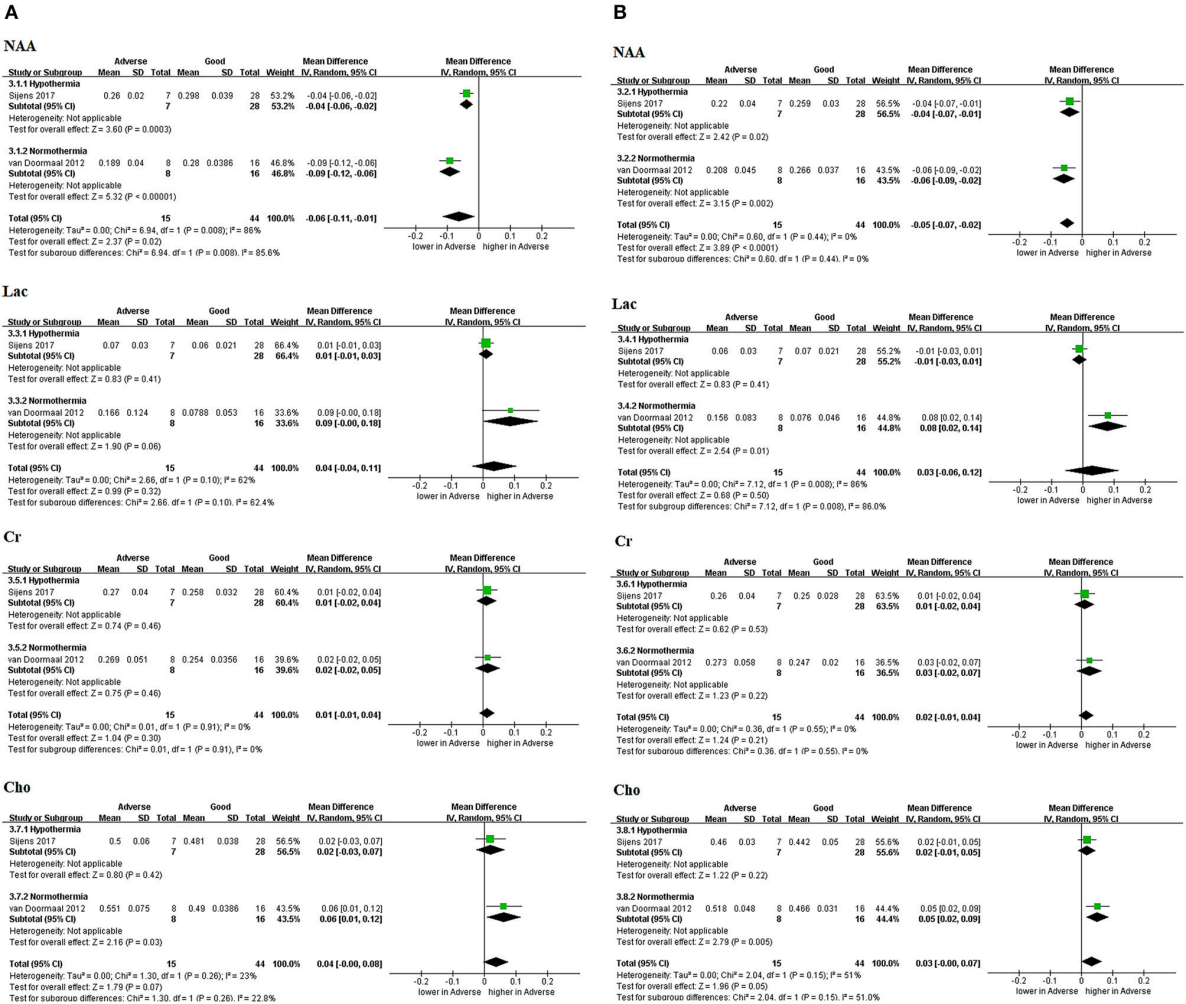
Graphic depiction of the results of meta-analyses: relative concentration in **(A)** white matter and **(B)** gray matter (NAA, Lac, Cr, and Cho). Relative concentration of metabolites means that metabolites were expressed in percent (%) of summed peak areas of Cho, Cr, and NAA in all the analyzed voxels. Cho, choline; Cr, creatine; Lac, lactate; NAA, N-acetylaspartate.

**Figure 5:**
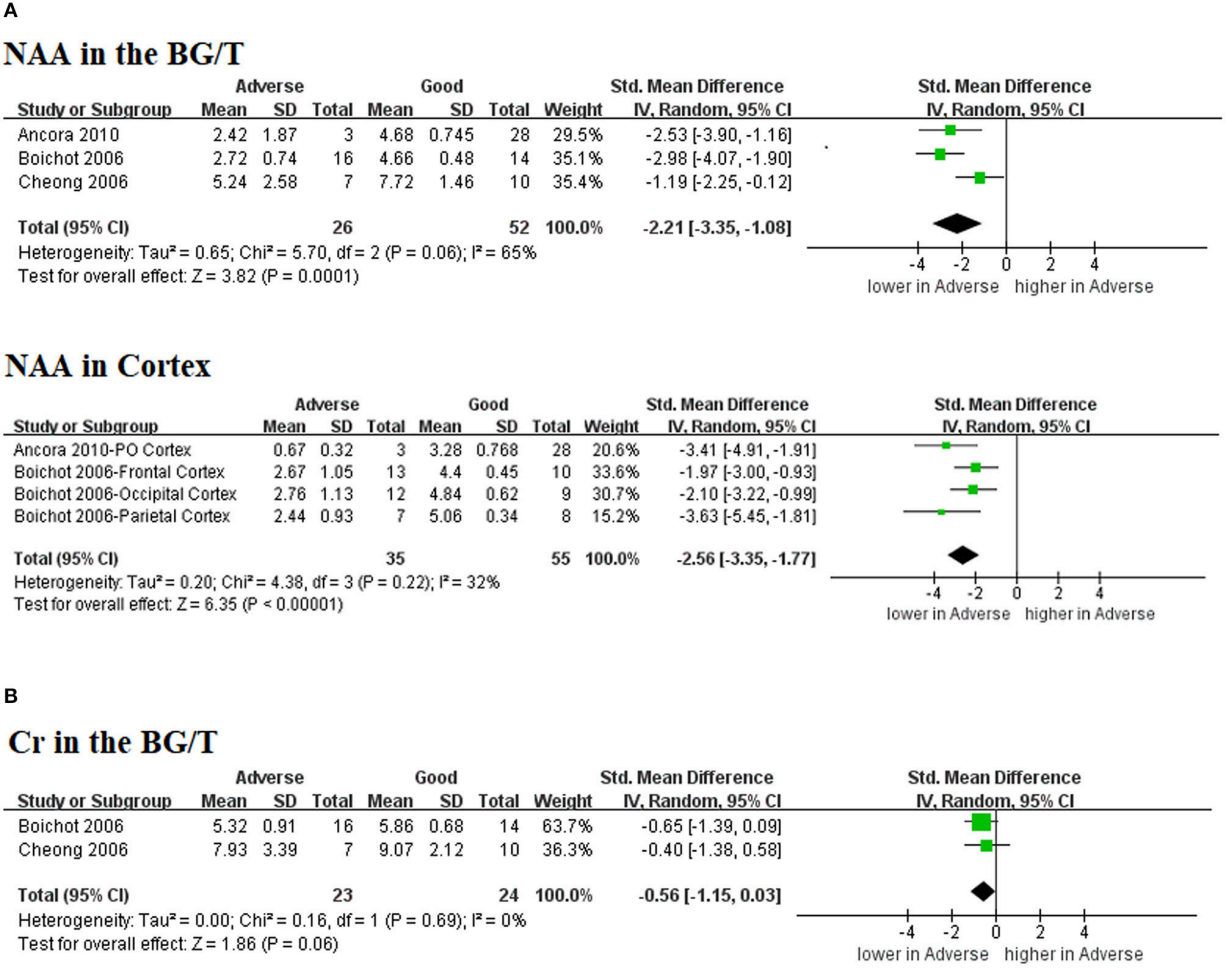
Graphic depiction of the results of meta-analyses: absolute concentration of **(A)** NAA in the BG/T and cerebral cortex, together with **(B)** Cr in the BG/T. BG/T, basal ganglia or thalamus; Cr, creatine; NAA, N-acetylaspartate.

The values of parameters, namely NAA/Cr, NAA/Cho, mI/Cr, mI/Cho, NAA, Lac, Cr, Cho, and mI data, were extracted. Sufficient data for the meta-analysis were acquired and subgroup analyses were conducted based on the use of TH whenever possible. The overall results of the meta-analysis are presented in Figures 3–**5**, respectively.

#### Peak area ratios

One study by Ancora et al. (20) performing ^1^H-MRS on neonates who underwent TH after rewarming (*n* = 20; prospective) was included in all the analyses of peak area ratios (Figures 3A,B).

Three peak area ratios revealed predictive values for adverse outcomes in TH subgroup and the combined results (with and without TH): NAA/Cr in BG/T in TH (MD −0.31, 95%CI −0.55 to −0.07, 1 study) and combined results (MD −0.37, 95% CI −0.49 to −0.25, 4 studies, I^2^ = 15%); NAA/Cho in BG/T in TH (MD −0.89, 95%CI −1.43 to −0.35, 1 study) and combined results (MD −0.25, 95%CI −0.42 to −0.07, 4 studies, I^2^ = 74%); and mI/Cho in cerebral cortex in TH (MD −1.94, 95%CI −3.69 to −0.19, 1 study) and combined results (MD −1.64, 95%CI −2.64 to −0.64, 2 studies, I^2^ = 0%).

However, three peak area ratios revealed inconsistently predictive values between TH subgroup and the combined results: NAA/Cr in cerebral cortex in TH (MD −0.17, 95%CI −0.41 to 0.07, 1 study) and combined results (MD −0.46, 95%CI −0.68 to −0.23, 3 studies, I^2^ = 82%); NAA/Cho in cerebral cortex in TH (MD −0.28, 95%CI −0.89 to 0.33, 1 study) and combined results (MD −0.24, 95%CI −0.39 to −0.10, 4 studies, I^2^ = 77%). And mI/Cr in BG/T in TH (MD −0.48, 95%CI −0.72 to −0.24, 1 study) and combined results (MD −0.27, 95%CI −0.68 to 0.14, 2 studies, I^2^ = 84%); as well as in cerebral cortex in TH (MD −0.77, 95%CI −1.49 to −0.05, 1 study) and combined results (MD −0.47, 95%CI −1.39 to 0.45, 2 studies, I^2^ = 33%).

In addition, mI/Cho exhibited no predictive value in BG/T in TH (MD −0.73, 95%CI −1.96 to 0.50, 1 study) and combined results (MD −0.38, 95%CI −1.01 to 0.26, 2 studies, I^2^ = 0%).

#### Relative concentration

In 2 of 7 studies (29%), relative concentration was used to measure parameters including NAA, Lac, Cr, and Cho (16, 17), and one study by Sijens et al. (16) was performed after TH (*n* = 35; retrospective). The sub-group was divided into WM and GM based on the tissue type contained in the ROIs (Figures 4A,B). These were not combined with absolute quantification because they were a comparison between the relative levels of specific metabolites. NAA was the only biomarker that demonstrated predictive value in WM in TH (MD −0.04, 95%CI −0.06 to −0.02, 1 study) and combined results (MD −0.06, 95%CI −0.11 to −0.01, 2 studies, I^2^ = 86%); in GM in TH (MD −0.04, 95%CI −0.07 to −0.01, 1 study) and combined results (MD −0.05, 95%CI −0.07 to −0.02, 2 studies, I^2^ = 0%).

#### Absolute concentration

Three (43%) studies (21–23) reported the absolute concentration of metabolites, while none were performed in the era of TH (Figure 5). Only NAA demonstrated a significantly decreased level in neonates with adverse outcomes as shown in Figure 5A (SMD −2.21, 95% CI −3.35 to −1.08, 3 studies, I^2^ = 65% in the BG/T; SMD −2.56, 95% CI −3.35 to −1.77, 2 studies, I^2^ = 32%). Cr in the BG/T was constant between groups (SMD −0.56, 95% CI −1.15 to 0.03, 2 studies, I^2^ = 0%) (Figure 5B). Meta-analyses were not conducted for the remaining metabolites due to insufficient data.

### Sensitivity analysis

A sensitivity analysis for NAA/Cr level revealed no unstable results in both sub-regions, while for NAA/Cho in the BG/T, the predictive value was lost when the study by Boichot et al. (22) or Cheong et al. (23) was removed. Meanwhile, there was a significant reduction in heterogeneity of NAA/Cho in the cerebral cortex when the study by Ancora et al. (21) was excluded. Regarding the absolute concentration of NAA in the BG/T, the heterogeneity of the result was lower after removing the study by Cheong et al. (23).

## Discussion

To our knowledge, this was the first systematic review to investigate all three types of ^1^H-MRS biomarkers as predictors of adverse outcomes in term neonates with perinatal asphyxia and HIE in the era of TH. The results demonstrate the association of several markers with adverse outcomes after adding results from hypothermic neonates, not restricted to peak area ratios alone. Nevertheless, the study has a few limitations that must be addressed before discussing the predictive values of the identified biomarkers. First, our results were largely based on a small sample size, and only two small studies have been conducted in cooled babies. Therefore, larger prospective multicenter studies are required. Second, the studies being compared were not uniform because of the differences in follow-up time, the tools for outcome assessment, the age range at which ^1^H-MRS was performed, and the parameters of the ^1^H-MRS equipment. Therefore, the present findings may demonstrate a substantial level of statistical heterogeneity (Figure S1). Third, although the I-squared parameter is less dependent on the number of studies as *Q*-test, the results still need to be interpreted with caution based on the small groups of studies. Finally, we used MD or SMD to calculate the capability of discrimination between different outcomes, instead of calculating the sensitivity and specificity with diagnostic thresholds. Hence, our results only suggest whether a marker was statistically significant as a constructive marker of adverse outcomes rather than comment on the true prognostic values, as previously reported (7).

Despite the limitations, the study yielded some enlightening findings, with sensitivity analyses emphasizing the robustness of our results. Three peak area ratios revealed potential predictive values for adverse outcomes in TH subgroup and combined results: NAA/Cr and NAA/Cho in the BG/T, and mI/Cho in cerebral cortex. Moreover, NAA relative concentration is associated with adverse outcomes in TH and combined results in both WM and GM. NAA absolute concentration also showed prognostic capability in normothermic neonates in BG/T and cerebral cortex. Therefore, NAA may be a potential marker in outcome prediction for HIE in the era of TH.

In addition, three peak area ratios revealed inconsistently predictive values between TH subgroup and combined results: NAA/Cr and NAA/Cho in cerebral cortex, as well as mI/Cr in BG/T and cerebral cortex. The discrepancy may be due to the different predictive value between TH and normothermic conditions. Also, it may be due to the small sample size of included studies.

The meta-analysis demonstrates that NAA/Cr has a power of prediction in both sub-regions, although there was significant heterogeneity among the studies in the cerebral cortex (I^2^ = 82%). In addition, the latent capability of NAA/Cho to predict outcomes must be cautiously interpreted because the heterogeneity was also substantial among the studies (I^2^ = 74% in the BG/T and I^2^ = 77% in the cortex). Moreover, the predictive value of NAA/Cho in the BG/T was lost when we removed the studies by Boichot et al. (22) or Cheong et al. (23), which also reflects the instability of NAA/Cho. NAA was also the only metabolite that was significantly different between the groups with diverse outcomes when compared according to concentration.

To our knowledge, NAA is found nearly exclusively in neurons, and it is considered to be a marker of neuronal maturation/integrity (23, 25). Previously, Cr and/or Cho have been used as a denominator in peak area ratios because they were considered to be stable in cases of hypoxia-ischemia (21, 22). However, the differences in prognostic abilities indicated by MD between NAA/Cr and NAA/Cho reveal that Cho may not constitute a stable reference for peak area ratios. For example, in the sub-region of the BG/T, NAA/Cr has higher a MD value with a narrower 95% CI, indicating a larger magnitude of effect when compared with NAA/Cho. This deduction is consistent with previous studies, in which the absolute concentration of Cho was different between groups with different outcomes (22, 23). According to the present findings, Cr demonstrated no significant difference between different outcome groups, neither in the analysis of studies on pure normothermic participants (Figure 5B) nor in that of studies on neonates who had undergone TH (Figures 4A,B). However, Cheong et al. (23) demonstrated changes in the absolute concentration of Cr between neonates with different neurodevelopmental outcomes. This discrepancy may be due to the limited ROI included as well as the small sample size in our meta-analysis. Whereas, NAA/Cr in the BG/T appeared to retain the ability as a potential prognostic biomarker after cooling based on the subgroup analysis (Figure 3A). Sensitivity analysis also revealed stable results for NAA/Cr level in BG/T. In contrast, the relative Cho concentration acted as a stable reference after TH, as shown in Figures 4A,B. Nevertheless, this inference is made on the basis of two studies conducted in the same center (16, 17), and more evidence is needed to corroborate this speculation. Nevertheless, the results should be cautiously interpreted when expressed relative to Cho.

Meanwhile, an increase in the relative concentration of NAA after TH was observed in WM, as shown in Figure 4A. There was also an echo, albeit somewhat weaker, in GM in the present study. This phenomenon may reflect the neuroprotection of TH on neurons. The discrepancy between different brain tissues is probably due to the different degree of severity in the tissue, as previously reported (26). NAA loss from WM is smaller than that in GM and is milder. In that case, the WM may have benefited more from TH. If so, the predictive value of NAA in the WM may weaken. Nevertheless, more evidence is needed before definitive conclusions can be drawn.

We did not acquire sufficient data for Lac/NAA for further analysis, although according to Alderliesten et al. (8), Lac/NAA in the BG/T retained its prognostic capability after TH with a higher cut-off value. Based on previous studies on TH, one of the speculated mechanisms of TH is that it would facilitate a more efficient cellular function with less lactate production by reducing the cerebral metabolic rate by 5–8% for every 1°C reduction in core temperature (27). This hypothesis was also consistent with the findings of Sijens et al. (16), as well as the results of our meta-analysis of the relative lactate concentration. Under this circumstance, lactate would, thus, be a less reliable predictive marker in the era of TH, unlike it used to be in untreated newborns. Moreover, a recent animal study has reported that the lactate content would become maximal at 2–6 h after the hypoxic-ischemic insult and then gradually decreased to the level of the control group even in normothermic models (28). Considering the time window and the duration of TH, it is probable that the relative concentration of lactate would not be sufficiently prognostic when performing ^1^H-MRS scans. However, Thayyil et al. (5) found that Lac/NAA in the BG/T was an accurate MR biomarker for prediction of outcome in children with HIE. These differences may be because a very few lactate measurements recommended by Thayyil et al. were included in our meta-analysis. It may also be due to the possible influences of TH that decreased the lactate production (27). Additionally, the difference of measuring methods and ROIs leads to the different findings between our study and Thayyil's meta-analysis.

With regard to the mI content, we cannot draw a definite conclusion that mI/Cho in the cortex can serve as a good predictor because of its wide 95% CI and limited data.

Based on our findings, NAA concentration and NAA/Cr in the BG/T region were recommended to predict the outcomes of HIE. We did notice that the metabolite's spectral appearances evolve with the echo time (TE), for example, lactate is completely inverted at TE of 144 ms and fully upright at 288 ms (29). The choice of TE is also dependent on what metabolites that investigators are interested in (30). In addition, TE affects peak amplitude owing to relaxation effects which will vary between metabolites. Furthermore, for coupled spin systems using standard point-resolved spectroscopic sequence localization methods, there is a chemical shift displacement artifact which usually leads to partial signal cancellation and an apparently lower signal than should be the case. Therefore, the consistency in acquisition protocols as well as in analysis is important and necessary (31). However, no direct comparison was performed in the same study regarding the effects of different TE on the outcome measurements of HIE neonates. Further studies are required to conduct an optimized standard protocol for both measurement protocols and analysis methods.

Two previous meta-analyses evaluated ^1^H-MRS on its predictive value of neurodevelopmental outcomes in HIE before the era of TH. Thayyil et al. (5) considered BG/T Lac/NAA to be the most accurate MR biomarker, while van Laerhoven et al. (6) reported that the evidence to inform the use of ^1^H-MRS is insufficient. Based on our analysis, the role of lactate after TH needs to be reconsidered and more prospective studies need to be performed.

## Conclusions

The present study adds to existing knowledge by suggesting that attention be devoted to the predictive value of the content of NAA, particularly in the sub-region of the BG/T. It seems that MDs for the ratios including NAA are larger than for its relative concentration, and therefore are more likely to be measurable in a clinical context. Furthermore, NAA/Cr in the BG/T could be a potential prognostic biomarker to evaluate neurodevelopmental outcomes. In addition, there may be issues with the prognostic ability of lactate due to the hypothermia treatment. As only two small studies have been conducted under TH, larger prospective multicenter studies with a standardized protocol for both measurement protocols and analysis methods are required in future studies.

## Author contributions

RZ, TX, and DM conceived and designed the review. RZ, TX, LZ, SL, FZ, YT, YQ, and DM coordinated the review. RZ, TX, YQ, and DM designed search strategies. RZ and TX extracted data. RZ, TX, LZ, SL, FZ, YT, YQ, and DM wrote the review. DM provided general advice on the review. DM revised the manuscript and was the fund contributor.

### Conflict of interest statement

The authors declare that the research was conducted in the absence of any commercial or financial relationships that could be construed as a potential conflict of interest.
