# Supplementary material for: Proton Magnetic Resonance Spectroscopy Biomarkers in Neonates With Hypoxic-Ischemic Encephalopathy: A Systematic Review and Meta-Analysis
Source: Front Neurol. 2018 Aug 31;9:732. doi: 10.3389/fneur.2018.00732 (PMC6127251; doi:10.3389/fneur.2018.00732)
Supplement: Supplementary file 1 [file Table_1.doc]

Supplementary Material

**Proton Magnetic Resonance Spectroscopy Biomarkers in Neonates with Hypoxic-Ischemic Encephalopathy: A Systematic Review and Meta-analysis**

**Rong Zou^1,2#^, Tao Xiong^1,2#^, Li Zhang^1,2^, Shiping Li^1,2^, Fengyan Zhao^1,2^, Yu Tong^1,2^, Yi Qu^1,2^, Dezhi Mu^1,2*^**

^1^Department of Pediatrics, West China Second University Hospital, Sichuan University, Chengdu, China.

^2^Key Laboratory of Birth Defects and Related Diseases of Women and Children, Ministry of Education, Sichuan University, Chengdu, China.

^#^ These authors contributed equally to this report.

*** Correspondence:**

Dezhi Mu

mudz@scu.edu.cn

**Table S1 Modified QUADAS-2 tool for evaluation of methodological quality.**

| **Domain 1: Patient selection**  **Ideal: A consecutive or a random sample with suspected disease** |
| --- |
| **Risk of Bias**: Could the selection of patients have introduced bias?  -Firstly, the method of people selection will be recorded, and then signaling questions 1 and 2 will be answered as "yes", "no" or "unclear".  -The risk of bias will be judged as "low" if both signaling questions 1 and 2 are answered "yes". The risk of bias will be judged as "high" if either signaling question is answered "no". The risk of bias will be judged as "unclear" if there are insufficient data to permit a judgment.  **1. Was a consecutive or random sample of patients enrolled?**  YES if the study specifically states that consecutive patients or a random sample was selected.  NO if the study clearly states that the selection of patients was not consecutive or random, or if this can be easily inferred from the design.  UNCLEAR if not reported or cannot be determined.  **2. Did the study avoid inappropriate exclusions?**  YES: If the study population was selected from neonates diagnosed with having perinatal asphyxia and hypoxic-ischemic encephalopathy with no co-morbidities AND if patients were not excluded based on any finding at clinical examination or other diagnostic imaging.  NO: If the study population included neonates without diagnoses of perinatal asphyxia and hypoxic-ischemic encephalopathy or with other diseases of similar pathological changes OR if patients were excluded based on any finding at clinical examination or other diagnostic imaging. |
| **Is there concern that the included patients do not match:**  Overall assessment of level of concern regarding applicability  "low" if no major concerns; "high" if any major concerns; "unclear" if not enough information to judge.  Major concern: If a study included patients with other diseases of similar pathological changes.  Major concern: If a study included perinatal asphyxia patients without evidence of encephalopathy. |

| **Domain 2: Index test Proton Magnetic Resonance Spectroscopy (^1^H-MRS)** |
| --- |
| **Risk of Bias:**  Could the conduct or interpretation of the index tests have introduced bias?  -Firstly, the imaging parameters of ^1^H-MRS will be recorded, as well as how it was conducted and interpreted, and then two signaling questions will be answered as "yes", "no" or 'unclear".  -The risk of bias will be judged as "low" if both the signaling questions are answered "yes". The risk of bias will be judged as "high" if either signaling question is answered "no". The risk of bias will be judged as "unclear" if there are insufficient data to permit a judgment.  **1. Were the index tests results interpreted without knowledge of the results of neurodevelopmental outcomes?**  YES if the study states that the interpretation of the index test was blinded to the follow-up outcomes.  NO if the study clearly states that interpretation of the index test was not blinded to the follow-up outcomes.  UNCLEAR if not reported or cannot be determined  **2. If a threshold was used, was it pre-specified?**  YES if the threshold used to define adverse/good outcome was pre-specified.  NO if the thresholds used to define adverse/good outcome was derived from the results of the study.  UNCLEAR if not reported or cannot be determined. |
| **Are there concerns that the index test, its conduct, or interpretation differ from the review question:**  Overall assessment of level of concern regarding applicability  "low" if no major concerns; "high" if any major concerns; "unclear" if not enough information to judge.  Major concern: ^1^H-MRS protocol varies between patients or differs significantly from other studies included in the sample. |

| **Domain 3: Reference standard** |
| --- |
| **Risk of Bias:** Could the reference standard, its conduct or its interpretation have introduced bias?  The information of outcome assessments will also be recorded and used to judge if the method used for follow-up can be used to assess long-term outcomes of patients with hypoxic-ischemic encephalopathy.  The risk of bias will be judged as "low" if both the signaling questions are answered "yes". The risk of bias will be judged as "high" if either signaling question is answered "no". The risk of bias will be judged as "unclear" if there are insufficient data to permit a judgment.  **1. Is the reference standard likely to correctly classify the target condition?**  YES If at least 1 of the following 3 items are used as outcome measures together with survival: impairment, perceived disability in physical activities or performance in physical activities, quality of life.  NO If the study did not perform any of the following 3 items to measure the outcome in addition to survival: impairment, perceived disability in physical activities or performance in physical activities, quality of life.  UNCLEAR if not reported or cannot be determined.  **2. Were the reference standard results interpreted without knowledge of the index test?**  YES if the study clearly states that the index test was not used as part of the reference criteria AND the assessment of the reference criteria was blinded to the index test result.  NO if the study clearly states that the index test was used as part of the reference criteria OR the assessment of the reference criteria was not blinded to the index test result.  UNCLEAR if not reported or cannot be determined. |
| **Are there concerns that the target condition as defined by the reference standard does not match the question:**  Overall assessment of level of concern regarding applicability  "low" if no major concerns; "high" if any major concerns; "unclear" if not enough information to judge.  Major concern: significantly different reference standard compared to other studies. |

| **Domain 4: Timing and flow** |
| --- |
| **Risk of Bias:** Could the patient flow have introduced bias?  -Firstly, the patients who were excluded from the final analysis will be recorded, and then signaling questions 3 will be answered as "yes", "no" or "unclear".  -The risk of bias will be judged as "low" if all the signaling question are answered "yes". The risk of bias will be judged as "high" if any signaling question is answered "no". The risk of bias will be judged as "unclear" if there are insufficient data to permit a judgment.  **1. Was there an appropriate time interval between the index test and reference standard?**  The reference standard involves follow-up for at least 12 months.  **2. Did all patients receive the same reference standard?**  Studies are eligible for the meta-analysis only if patients underwent outcome measures after at least 12 months follow up independent of the ^1^H-MRS results.  **3. Were all patients included in the analysis?**  YES The number of patients enrolled is equal to the number of patients included in the results.  NO The number of patients enrolled differs from to the number of patients included in the results.  UNCLEAR if not reported or cannot be determined. |
