# Supplementary material for: Proton Magnetic Resonance Spectroscopy Biomarkers in Neonates With Hypoxic-Ischemic Encephalopathy: A Systematic Review and Meta-Analysis
Source: Front Neurol. 2018 Aug 31;9:732. doi: 10.3389/fneur.2018.00732 (PMC6127251; doi:10.3389/fneur.2018.00732)

Supplementary Material

Proton Magnetic Resonance Spectroscopy Biomarkers in Neonates with Hypoxic-Ischemic Encephalopathy: A Systematic Review and Meta-analysis

Rong Zou[1,2#], Tao Xiong[1,2#], Li Zhang[1,2], Shiping Li[1,2], Fengyan Zhao[1,2], Yu Tong[1,2], Yi Qu[1,2], Dezhi Mu[1,2*]

[1]Department of Pediatrics, West China Second University Hospital, Sichuan University, Chengdu, China.

[2]Key Laboratory of Birth Defects and Related Diseases of Women and Children, Ministry of Education, Sichuan University, Chengdu, China.

[#] These authors contributed equally to this report.

*** Correspondence:**

Dezhi Mu

mudz@scu.edu.cn

Supplementary Figure 1. Graphic depiction of the results of meta-analyses including studies with follow up > 18 months: Peak area ratios in the (A) BG/T and (B) cerebral cortex (NAA/Cr, NAA/Cho, mI/Cr, and mI/Cho). BG/T, basal ganglia or thalamus; Cho, choline; Cr, creatine; mI, myo-Inositol; NAA, N-acetylaspartate.

A

NAA/Cr

Study or Subgroup	Adverse Mean	Adverse SD	Adverse Total	Good Mean	Good SD	Good Total	Weight	Mean Difference IV, Random, 95% CI
Ancora 2010	0.47	0.36	3	0.85	0.102	28	25.8%	-0.38 [-0.79, 0.03]
Ancora 2013	0.46	0.3	6	0.77	0.05	14	74.2%	-0.31 [-0.55, -0.07]
**Total (95% CI)**			**9**			**42**	**100.0%**	**-0.33 [-0.54, -0.12]**

Heterogeneity: Tau² = 0.00; Chi² = 0.08, df = 1 (P = 0.77); I² = 0%
Test for overall effect: Z = 3.09 (P = 0.002)

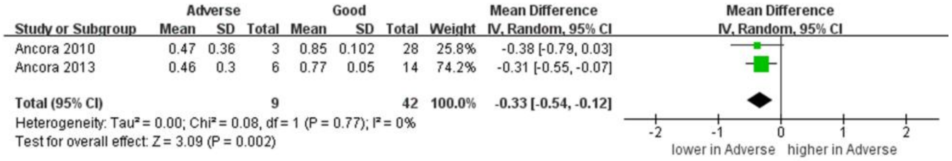

NAA/Cho

Study or Subgroup	Adverse Mean	Adverse SD	Adverse Total	Good Mean	Good SD	Good Total	Weight	Mean Difference IV, Random, 95% CI
Ancora 2010	1.3	1.03	3	2.265	0.344	28	17.3%	-0.97 [-2.14, 0.21]
Ancora 2013	1.18	0.65	6	2.07	0.25	14	82.7%	-0.89 [-1.43, -0.35]
**Total (95% CI)**			**9**			**42**	**100.0%**	**-0.90 [-1.39, -0.42]**

Heterogeneity: Tau² = 0.00; Chi² = 0.01, df = 1 (P = 0.91); I² = 0%
Test for overall effect: Z = 3.63 (P = 0.0003)

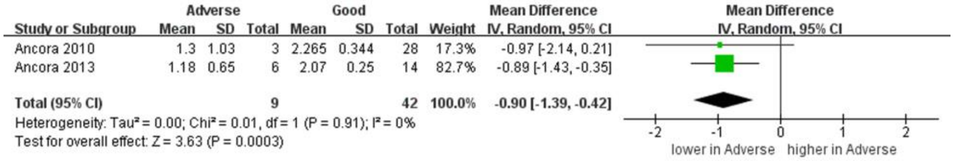

mI/Cr

Study or Subgroup	Adverse Mean	Adverse SD	Adverse Total	Good Mean	Good SD	Good Total	Weight	Mean Difference IV, Random, 95% CI
Ancora 2010	0.9	0.19	3	0.96	0.197	28	50.4%	-0.06 [-0.29, 0.17]
Ancora 2013	0.46	0.28	6	0.94	0.15	14	49.6%	-0.48 [-0.72, -0.24]
**Total (95% CI)**			**9**			**42**	**100.0%**	**-0.27 [-0.68, 0.14]**

Heterogeneity: Tau² = 0.07; Chi² = 6.28, df = 1 (P = 0.01); I² = 84%
Test for overall effect: Z = 1.28 (P = 0.20)

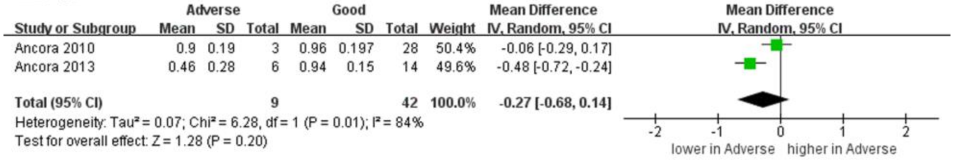

mI/Cho

Study or Subgroup	Adverse Mean	Adverse SD	Adverse Total	Good Mean	Good SD	Good Total	Weight	Mean Difference IV, Random, 95% CI
Ancora 2010	2.3	0.64	3	2.55	0.378	28	73.6%	-0.25 [-0.99, 0.49]
Ancora 2013	1.56	1.51	6	2.29	0.46	14	26.4%	-0.73 [-1.96, 0.50]
**Total (95% CI)**			**9**			**42**	**100.0%**	**-0.38 [-1.01, 0.26]**

Heterogeneity: Tau² = 0.00; Chi² = 0.43, df = 1 (P = 0.51); I² = 0%
Test for overall effect: Z = 1.17 (P = 0.24)

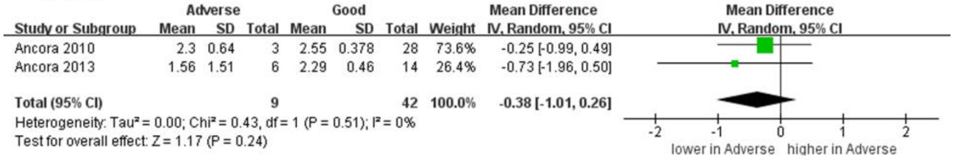

B

NAA/Cr

Study or Subgroup	Adverse Mean	Adverse SD	Adverse Total	Good Mean	Good SD	Good Total	Weight	Mean Difference IV, Random, 95% CI
Ancora 2010-PO Cortex	0.16	0.15	3	0.965	0.172	28	50.8%	-0.80 [-0.99, -0.62]
Ancora 2013-PO Cortex	0.69	0.29	6	0.86	0.1	14	49.2%	-0.17 [-0.41, 0.07]
**Total (95% CI)**			**9**			**42**	**100.0%**	**-0.49 [-1.11, 0.13]**

Heterogeneity: Tau² = 0.19; Chi² = 17.32, df = 1 (P < 0.0001); I² = 94%
Test for overall effect: Z = 1.55 (P = 0.12)

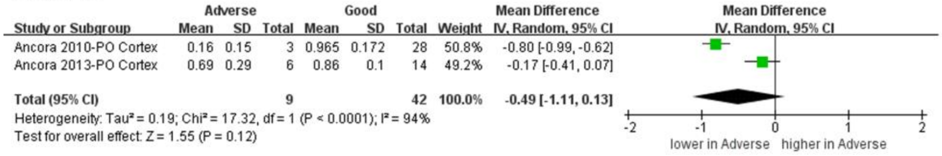

NAA/Cho

Study or Subgroup	Adverse Mean	Adverse SD	Adverse Total	Good Mean	Good SD	Good Total	Weight	Mean Difference IV, Random, 95% CI
Ancora 2010-PO Cortex	0.87	0.51	3	2.3	0.417	28	30.9%	-1.43 [-2.03, -0.83]
Ancora 2013-PO Cortex	1.77	0.74	6	2.05	0.31	14	30.6%	-0.28 [-0.89, 0.33]
Kadri 2003-Occipital GM	0.505	0.2	14	0.691	0.207	19	38.5%	-0.19 [-0.33, -0.05]
**Total (95% CI)**			**23**			**61**	**100.0%**	**-0.60 [-1.33, 0.13]**

Heterogeneity: Tau² = 0.35; Chi² = 15.80, df = 2 (P = 0.0004); I² = 87%
Test for overall effect: Z = 1.61 (P = 0.11)

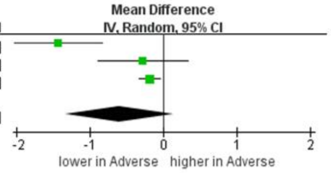

mI/Cr

Study or Subgroup	Adverse Mean	Adverse SD	Adverse Total	Good Mean	Good SD	Good Total	Weight	Mean Difference IV, Random, 95% CI
Ancora 2010-PO Cortex	2.68	1.31	3	2.42	0.356	28	29.1%	0.26 [-1.23, 1.75]
Ancora 2013-PO Cortex	1.49	0.85	6	2.26	0.43	14	70.9%	-0.77 [-1.49, -0.05]
**Total (95% CI)**			**9**			**42**	**100.0%**	**-0.47 [-1.39, 0.45]**

Heterogeneity: Tau² = 0.18; Chi² = 1.49, df = 1 (P = 0.22); I² = 33%
Test for overall effect: Z = 1.00 (P = 0.32)

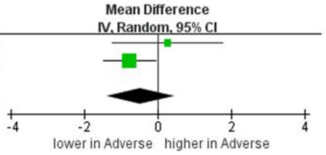

mI/Cho

Study or Subgroup	Adverse Mean	Adverse SD	Adverse Total	Good Mean	Good SD	Good Total	Weight	Mean Difference IV, Random, 95% CI
Ancora 2010-PO Cortex	4.34	1.03	3	5.835	0.906	28	67.6%	-1.50 [-2.71, -0.28]
Ancora 2013-PO Cortex	3.42	2.08	6	5.36	1.04	14	32.4%	-1.94 [-3.69, -0.19]
**Total (95% CI)**			**9**			**42**	**100.0%**	**-1.64 [-2.64, -0.64]**

Heterogeneity: Tau² = 0.00; Chi² = 0.17, df = 1 (P = 0.68); I² = 0%
Test for overall effect: Z = 3.22 (P = 0.001)

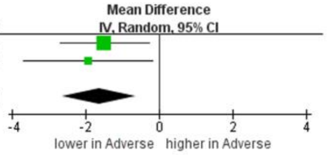

Supplement: Supplementary file 2 [file Image_1.pdf]
